# Combined Preimplantation Genetic Testing for Autosomal Dominant Polycystic Kidney Disease: Consequences for Embryos Available for Transfer

**DOI:** 10.3390/genes11060692

**Published:** 2020-06-24

**Authors:** Pere Mir Pardo, José Antonio Martínez-Conejero, Julio Martín, Carlos Simón, Ana Cervero

**Affiliations:** 1 Igenomix, 46980 Valencia, Spain; joseantonio.conejero@igenomix.com (J.A.M.-C.); julio.martin@igenomix.com (J.M.); Carlos.simon@igenomix.com (C.S.); ana.cervero@igenomix.com (A.C.); 2 Igenomix Foundation-INCLIVA, 46010 Valencia, Spain; 3 Department of Obstetrics and Gynecology, BIDMC, Harvard University, Boston, MA 02215, USA; 4 Department of Pediatrics, Obstetrics & Gynecology, University of Valencia, 46010 Valencia, Spain

**Keywords:** combined preimplantation genetic testing, Preimplantation genetic testing for monogenic disorders (PGT-M), Preimplantation genetic testing for aneuploidy assessment (PGT-A), Autosomal dominant polycystic kidney disease (ADPKD), male infertility, advanced maternal age, aneuploidy

## Abstract

Autosomal dominant polycystic kidney disease (ADPKD) is the most common hereditary kidney disease and presents with genetic and clinical heterogeneity. ADPKD can also manifest extra-renally, and seminal cysts have been associated with male infertility in some cases. ADPKD-linked male infertility, along with female age, have been proposed as factors that may influence the clinical outcomes of preimplantation genetic testing (PGT) for monogenic disorders (PGT-M). Large PGT for aneuploidy assessment (PGT-A) studies link embryo aneuploidy to increasing female age; other studies suggest that embryo aneuploidy is also linked to severe male-factor infertility. We aimed to assess the number of aneuploid embryos and the number of cycles with transferable embryos in ADPKD patients after combined-PGT. The combined-PGT protocol, involving PGT-M by PCR and PGT-A by next-generation sequencing, was performed in single trophectoderm biopsies from 289 embryos in 83 PGT cycles. Transferable embryos were obtained in 69.9% of cycles. The number of aneuploid embryos and cycles with transferable embryos did not differ when the male or female had the ADPKD mutation. However, a significantly higher proportion of aneuploid embryos was found in the advanced maternal age (AMA) group, but not in the male factor (MF) group, when compared to non-AMA and non-MF groups, respectively. Additionally, no significant differences in the percentage of cycles with transferable embryos were found in any of the groups. Our results indicate that AMA couples among ADPKD patients have an increased risk of aneuploid embryos, but ADPKD-linked male infertility does not promote an increased aneuploidy rate.

## 1. Introduction

Autosomal dominant polycystic kidney disease (ADPKD) is the most common hereditary kidney disease, with an estimated prevalence of 1:1000 to 1:2500 individuals [1]. ADPKD is a genetically heterogeneous disorder attributed to two main genes: *PKD1* (located at chromosome 16p13.3) and *PKD2* (located at chromosome 4q21–q23). Mutations in *PKD1* account for most (78–85%) ADPKD cases, but more than 1500 different mutations in *PKD1* or *PKD2* have been identified in patients with ADPKD [1,2]. In addition, six pseudogenes with a high homology to *PKD1* have been identified on chromosome 16. Consequently, only 3.5 kb of the 14 kb of the *PKD1* transcript is single copy [1,3].

Mutations in *PKD1* generally cause more severe nephropathy than mutations in *PKD2*, and the age of onset varies within and between families. ADPKD is clinically heterogeneous, but generally characterized by cysts that develop in one or both kidneys and increase in number over time, leading to nephromegaly and chronic renal failure [1]. ADPKD can also have extra-renal manifestations, with cysts in the liver (70–90%), the seminal tract (10–35%), and more rarely in the pancreas (5–10%), as well as with non-cystic manifestations, such as intracranial aneurysms (9–12%) [1,2,3]. The presence of cysts in seminal vesicles and ejaculatory ducts may be associated with infertility or subfertility [4,5], though not always [6]. On the other hand, fertility does not appear to be affected in women with ADPKD mutations, but pregnant women with a compromised kidney function must be monitored for the development of complications such as hypertension and preeclampsia, which could affect reproductive outcomes [5].

The offspring of individuals affected by ADPKD have a 50% chance of inheriting the causal mutation. To avert the inheritance of the disorder, either prenatal (PND) or preimplantation genetic testing for monogenic disorders (PGT-M) can be offered [5,6,7,8,9]. PND uses DNA obtained from the fetus, usually collected from amniotic fluid or chorionic villus sampling, to inform medical termination of the pregnancy in cases with confirmed genetic abnormality. In contrast, regardless of the fertility status, PGT-M requires assisted reproductive techniques (ART) such as in-vitro fertilization (IVF) or intracytoplasmic sperm injection to assess embryonic DNA, and only genetically normal embryos for the analyzed condition will be candidates for transfer to the maternal uterus. This difference, combined with the variability in the expression of the disease and age of onset, means that most patients would prefer PGT-M over PND [5,7,10].

PGT-M is most often performed via a polymerase chain reaction (PCR) [11] to directly amplify specific DNA sequences from an embryo biopsy, as is applied to ADPKD couples [7,8,12]. While next-generation sequencing (NGS) has been clinically applied for PGT-M [13], this method has not yet been used for ADPKD cases. In familial ADPKD, the genetic and clinical heterogeneity can obscure the causal mutation in some cases, but a family history of the disease is known in one, two, or even three different generations [14]. This phenomenon facilitates performing PGT-M by PCR through linkage analysis [2,7,12]. This strategy can be complemented with an analysis of the specific mutation, but is not necessarily applied for familial ADPKD cases.

A recent paper successfully applied PGT-M for ADPKD in day-3 embryo biopsies, resulting in 27 healthy live births. The report also evaluated the impact of parental ADPKD on the clinical outcome, showing a lower clinical pregnancy rate and live birth rate in couples with the male partner affected compared with couples with the female partner affected [12]. However, the authors concluded that their multivariate logistic regression analysis only identified an association of an increased maternal age with a lower live birth rate [12]. Embryo aneuploidy rates are widely reported to increase with increasing female age [15,16,17] and to cause a lower pregnancy and delivery rate [17]. In contrast, the relationship between aneuploidy rates and male infertility factors is less clear; some studies found no difference in aneuploidy rates between male-factor (MF) infertility and normal sperm patients [18], while others only reported higher aneuploidy rates in severe infertility cases, suggesting that PGT aneuploidy assessment (PGT-A) may be advisable for these patients [19,20].

Increasingly, PGT-M and PGT-A are performed in the same embryo [21,22,23]. However, few instances of combined testing have been reported for ADPKD. Indeed, we identified only two reports of combined-PGT for ADPKD: a case report of two ART cycles from a woman with ADPKD in a couple with no reported fertility issues [8], and a series of seven cycles from seven couples with reported ADPKD-linked male infertility [24]. Importantly, combining PGT-A with PGT-M could reduce the number of transferable embryos (i.e., neither genetically abnormal nor aneuploid embryos would be considered for transfer) and may result in a higher number of ART cycles without a transferable embryo. To date, the data reported for combined-PGT in ADPKD are limited. With 37 embryos analyzed across the two reports, 18.2% (2/11) [8] and 46.2% (12/26) [24] of embryos were transferable.

To help guide clinical application, we aimed to ascertain the impact on the number of cycles with transferable embryos after introducing PGT-A into routine PGT-M for ADPKD. For this purpose, we assessed the percentages of aneuploid embryos, transferable embryos, and cycles with transferable embryos after combined-PGT in (i) overall ADPKD cases; (ii) cycles where the male carried the mutation compared to when the female carried the mutation; and (iii) patients grouped as those with an expected higher aneuploidy rate: advanced maternal age (AMA) or male-factor infertility (MF).

## 2. Materials and Methods

### 2.1. Patient Demography

This retrospective analysis included 74 couples affected by ADPKD that underwent at least one combined-PGT (PGT-M for ADPKD along with PGT-A for 24-chromosomes) from October 2016 to April 2020; nine of these couples underwent two combined-PGT cycles, for a total of 83 combined-PGT cycles. Most cases were associated with *PKD1* (83.1%) mutations, and the remainder (16.9%) with *PKD2* mutations. All 13 *PKD2* cases and 53/61 (86.4%) *PKD1* cases had a mutation identified.

Couples were ascertained from 39 IVF clinics in 10 countries. The top contributors were Spain (37 cases, 46 cycles), the USA (18 cases, 18 cycles), and Brazil (9 cases, 9 cycles).

### 2.2. Combined-PGT Protocol

All patients underwent genetic counseling and were requested to provide a report showing the causal mutation for ADPKD in any of the two progenitors or other family members, and/or a clinical report confirming the diagnosis of ADPKD before beginning PGT. Then, written informed consent for both PGT-M and PGT-A was obtained for all couples. Combined-PGT comprised four main steps: (1) PGT-M work-up; (2) an IVF procedure (controlled ovarian stimulation, ovum pick-up, fertilization, and embryo culture) to obtain trophectoderm biopsies from viable embryos; (3) the shipment of biopsies to our facilities; and (4) PGT-M and PGT-A protocols. Once the PGT-M work-up was approved by the staff of our company (Igenomix, Valencia, Spain), the IVF procedure was the responsibility of each IVF center. After the embryo biopsy, Igenomix handled the shipment of samples, analysis, and reporting. All trophectoderm biopsies were obtained on day 5 or 6 of embryo culture, and each biopsy was collected and shipped in a PCR tube containing 2 μL of phosphate-buffered saline as transport medium. Sample preparation for testing included whole genome amplification (WGA) using the Ion ReproSeq PGS Kit (ThermoFisher Scientific, Waltham, MA, USA). The same WGA product was used for both PGT-M and PGT-A. PGT-M was performed by PCR following a laboratory-developed protocol, and PGT-A was performed by a semi-automated protocol by NGS (ThermoFisher Scientific, Waltham, MA, USA).

### 2.3. PGT-M Work-Up

A preclinical work-up was required for all couples before undergoing PGT-M. Informativity for several polymorphic genetic markers (short tandem repeats (STR)) and segregation analysis were assessed for every family in genomic DNA from blood or buccal cells. Additionally, in cases in which the mutation in *PKD1* or *PKD2* was needed for PGT-M purposes, its analysis was also included in the work-up. Haplotyping analysis was assessed by fluorescence multiplex PCR using a minimum of 12 STR. Amplification was conducted using a T3000 thermocycler (Biometra, Goettingen, Germany) or similar equipment, and PCR products were analyzed in an AB3500 genetic analyzer (ThermoFisher Scientific, Waltham, MA, USA). After haplotyping analysis, a minimum of four informative STR, two upstream and two downstream, and all of them within 1–2 Mb, were required for approving the PGT-M work-up. The laboratory-developed protocol included the selected informative STR, as well as the *PKD* mutation when applicable.

### 2.4. PGT-M Protocol

PGT-M was performed on WGA products from trophectoderm biopsies using the same protocol selected in the PGT-M work-up. After PGT-M analysis, embryos were classified as (i) “normal”, when a paternal and a maternal haplotype were present, confirming the presence of the haplotype unlinked to the PKD mutation, and the mutation was not detected; (ii) “abnormal”, when a paternal and maternal haplotype were present, confirming the presence of the haplotype linked to the PKD mutation, and/or the mutation was detected; or (iii) “non-informative” or inconclusive results, when any paternal or maternal information was missing. For “non-informative” cases, it was not possible to specifically classify the embryo as normal or abnormal. In such cases, a trophectoderm re-biopsy was recommended, but clinics made the ultimate decision on whether to send a new biopsy.

### 2.5. PGT-A Protocol

For aneuploidy assessment, DNA barcoding was performed during the WGA process using the Ion ReproSeq PGS Kit (ThermoFisher Scientific, Waltham, MA, USA). Ion Chef equipment was used for library preparation, and samples were sequenced in batches of 24 or 96 samples (using 520 and 530 chips, respectively) in an S5 XL sequencer (ThermoFisher Scientific, Waltham, MA, USA), as previously described [25].

After PGT-A analysis, embryos were classified as (i) “euploid”, when no aneuploidy was found; (ii) “aneuploid”, when at least one aneuploidy was found; or (iii) “non-informative” or inconclusive, when the pattern obtained did not enable a definitive classification of the embryo as euploid or aneuploid. As for PGT-M, non-informative results prompted a recommendation for trophectoderm re-biopsy.

### 2.6. Combined-PGT Strategy

Only embryos both “normal” for PGT-M and “euploid” for PGT-A were considered to be “transferable” embryos. However, couples had several options in terms of electing a combined-PGT strategy: (i) to perform PGT-M and PGT-A in all embryos (elected for 29 cycles); (ii) to perform PGT-M first, and only apply PGT-A to embryos considered “normal” or “non-informative” (elected in 52 cycles); or (iii) to perform PGT-A first, and only apply PGT-M to embryos considered “euploid” or “non-informative” (elected in only two cycles). In total, PGT-M was performed in 432 embryos, PGT-A in 298 embryos, and combined-PGT in 289 embryos.

### 2.7. Analysis of Results

ADPKD results were divided into two groups, according to Berckmoes [12] classification, by whether the female (female-ADPKD) or the male (male-ADPKD) carried the ADPKD mutation. The main indicators analyzed were the (i) number of aneuploid embryos; (ii) number of transferable embryos; and (iii) number of cycles with transferable embryos after combined-PGT.

The same analysis was performed in patient subsets of AMA and MF, and compared with non-AMA and non-MF results, respectively. AMA was defined as 38 years old or above, and male patients with the ADPKD mutation that self-reported male infertility were considered for MF.

Statistical analyses for comparing the results of the different groups were conducted using Fisher’s exact test for categorical variables. Comparisons of mean ages were performed using an unpaired *t*-test. Statistical significance was defined by a two-sided test with a *p* value of < 0.05.

## 3. Results

Overall, in 69.9% of combined-PGT cycles (58/83), there was at least one transferable embryo. By PGT-M, 94.0% of these cycles (78/83) had genetically normal embryos; by PGT-A, 74.7% of cycles (62/83) had euploid embryos. Additionally, no difference in the percentage of cycles with transferable embryos was found between female-ADPKD and male-ADPKD (Table 1).

For combined-PGT, 98.2% of embryos were informative for both techniques. The percentage of transferable embryos tended to be higher in the ADPKD-male group, but did not reach statistical significance (43.8% vs. 33.8% in ADPKD-female; *p* = 0.0978). There were no differences in the number of euploid embryos (PGT-A) or the number of genetically normal ones (PGT-M) (Table 2).

Combined-PGT results in the subsets of AMA and MF couples showed no differences in the percentage of cycles with transferable embryos compared with the overall results (Table 3). However, the comparison of PGT-A results per embryo showed a significantly higher percentage of aneuploid embryos in the AMA group when compared with non-AMA cases (Table 4). In contrast, the percentage of aneuploid embryos in the MF group did not significantly differ from the percentage in non-MF cases (Table 5).

## 4. Discussion

This study provides detailed information to support the utility of combining PGT-M and PGT-A to guide embryo selection in couples affected by ADPKD who are undergoing ART. A larger study on PGT-M for ADPKD [12] reported 78 cycles and found that 35.7% of embryos were transferable (174/487), while 92.3% (72/78) of cycles had transferable embryos. These data can be compared with ours when only considering the PGT-M results; we found that 49.3% of embryos were transferable and 94.0% of cycles had transferable embryos. In our study, fewer cycles had transferable embryos after combined-PGT (94.0% of cycles with PGT-M vs. 69.9% with combined-PGT; *p* = 0.0001). This finding cannot be directly compared with previous publications due to limited data availability [8,24]. The percentage of transferable embryos we found after combined-PGT (39.1%) could be compared, but existing reports utilized small samples (2/11 or 18.2% [8]; 12/26 or 46.2% [24]).

Berckmoes et al. [12] suggested ADPKD-linked male infertility and female age as factors influencing the clinical outcome of PGT-M. In our data, when dividing cycles by male-ADPKD and female-ADPKD, results at the embryo level showed a similar percentage of (i) euploid embryos, (ii) genetically normal embryos, and (iii) transferable embryos after combined-PGT. Accordingly, no differences in the percentage of cycles with transferable embryos were detected between groups. Similarly, no differences were found when comparing MF versus non-MF cases. In contrast, the percentage of aneuploid embryos was significantly higher in the AMA group compared to the non-AMA group, but the number of cycles with transferable embryos was not statistically different.

In our study, ADPKD-linked male infertility was not associated with a higher aneuploidy rate or fewer cycles with transferable embryos; this finding was consistent across all male-ADPKD couples. Therefore, our data do not support that aneuploidy can be an influencing factor for poorer reproductive outcomes in male-ADPKD cases. These data align with recent reports on PGT-A results for MF patients, where significantly higher aneuploidy rates were only observed in severe cases [19,20], and not in overall MF patients [18]. However, in the present study, it was not possible to stratify the results by the severity of sperm parameters and, given the low number of cases of self-reported male infertility, any conclusions obtained for this group are limited.

On the other hand, AMA is established as a factor for higher aneuploidy rates [15,16,17]. Our data align with this phenomenon, and support the lower live birth rate associated with female age in Berckmoes et al. [12]. This higher aneuploidy rate will translate into a higher probability of transferring aneuploid embryos when PGT-A is not performed. For instance, aneuploid embryos still develop to the blastocyst stage and are responsible for lower probabilities of pregnancy, but a higher miscarriage likelihood [26]. Unfortunately, our study did not have access to data for pregnancy, miscarriage, or live birth rates, and Berckmoes et al. did not analyze PGT-M outcomes from the subset of patients categorized as AMA. However, in their data (see Table 2), the mean female age is significantly higher in the group of male-ADPKD compared with the female-ADPKD. Therefore, considering the information above, this feature may have had a negative impact on PGT-M outcomes for the male-ADPKD group.

## 5. Conclusions

In summary, we demonstrated a feasible approach for every ADPKD patient through a simultaneous analysis of PGT-M and PGT-A in the same embryo biopsy. The incorporation of combined-PGT helps in selecting euploid and genetically normal embryos, ensuring 70% of cycles with transferable embryos, and highlighting the impact of maternal age on embryo aneuploidy in this cohort. A similar approach could be available for every PGT-M case in the daily clinical routine.

## Figures and Tables

**Table 1:** Results per cycle presented by cases where the female (female-autosomal dominant polycystic kidney disease (ADPKD)) or the male (male-ADPKD) carried the ADPKD mutation.

	Male-ADPKD	Female-ADPKD	Total	*p*-Value
No. couples	41	33	74	-
No. combined-PGT cycles	47	36	83	-
Female age, years (SD)	36.3 (3.9)	35.8 (4.3)	36.1 (4.1)	ns
Mean no. analyzed embryos per cycle (SD)	5.4 (3.4)	5.4 (3.3)	5.4 (3.4)	ns
Cycles with transfer, *n* (%)	31 (66.0)	27 (75.0)	58 (69.9)	ns
% cycles with euploid embryos	70.2	80.6	74.7	ns
% cycles with PGT-M normal embryos	95.7	91.7	94.0	ns

ns: not significant; SD: standard deviation.

**Table 2:** Results per embryo presented by male-ADPKD and female-ADPKD cycles (% (n)).

EMBRYOS		Male-ADPKD	Female-ADPKD	TOTAL	*p*-Value
Combined PGT (*n* = 289)	Normal	43.8 (67)	33.8 (46)	39.1 (113)	ns
	Abnormal	54.2 (83)	64.7 (88)	59.2 (171)	
	NI	2.0 (3)	1.5 (2)	1.8 (5) *	
PGT-M (*n* = 432)	Normal	49.0 (123)	49.7 (90)	49.3 (213)	ns
	Abnormal	48.2 (121)	45.9 (83)	47.2 (204)	
	NI	2.8 (7)	4.4 (8)	3.5 (15)	
PGT-A (*n* = 298)	Normal	51.8 (84)	49.3 (67)	50.7 (151)	ns
	Abnormal	45.7 (74)	48.5 (66)	47.0 (140)	
	NI	2.5 (4)	2.3 (3)	2.3 (7)	

NI: non-informative embryos; ns: not significant; * final non-informative embryos. Seven embryos (three embryos in the male group and four in the female group) showed the presence of only one allele that was confirmed to be a monosomy for the corresponding chromosome after preimplantation genetic testing aneuploidy assessment (PGT-A) analysis. Therefore, they were initially classified as non-informative for PGT for monogenic disorders (PGT-M), but finally classified as chromosomally abnormal.

**Table 3:** Results per cycle in the advanced maternal age (AMA) and male-factor infertility (MF) groups.

	AMA	MF	OVERALL	*p*-Value
No. couples	23	5	74	
No. combined-PGT cycles	32	6	83	
Female age, years (SD)	39.5 (1.3)	33.3 (2.2)	36.1 (4.1)	<0.0001 *
Mean no. analyzed embryos (SD)	4.9 (3.2)	5.2 (3.8)	5.4 (3.4)	ns
Cycles with euploid embryos, *n* (%)	20 (62.5)	5 (83.3)	62 (74.7)	ns
Combined-PGT cycles with transfer, *n* (%)	18 (56.3)	4 (66.7)	58 (69.9)	ns

ns: not significant; SD: standard deviation; * between AMA and MF.

**Table 4:** Comparison of results per embryo in AMA and non-AMA groups [% (n)].

		AMA	Non-AMA	*p*-Value
Combined PGT	Normal	28.0 (26)	44.4 (87)	ns
	Abnormal	69.9 (65)	54.1 (106)	
	NI	2.1 (2)	1.5 (3)	
PGT-M	Normal	46.1 (71)	50.4 (142)	ns
	Abnormal	47.4 (73)	47.8 (131)	
	NI	6.5 (10)	1.8 (5)	
PGT-A	Normal	33.3 (31)	58.5 (120)	<0.0001
	Abnormal	64.5 (60)	39.0 (80)	
	NI	2.2 (2)	2.5 (5)	

NI: non-informative embryos; ns: not significant.

**Table 5:** Comparison of results per embryo in MF and non-MF groups (% (n)).

		MF	Non-MF	*p*-Value
Combined PGT	Normal	64.7 (11)	37.5 (102)	
	Abnormal	25.3 (6)	60.7 (165)	ns
	NI	0	1.8 (5)	
PGT-M	Normal	54.8 (17)	48.6 (195)	
	Abnormal	45.2 (14)	47.6 (191)	ns
	NI	0	3.8 (15)	
PGT-A	Normal	70.6 (12)	49.5 (139)	
	Abnormal	29.4 (5)	48.0 (135)	ns
	NI	0	2.5 (7)	

NI: non-informative embryos; ns: not significant.

## References

[1] Cornec-Le Gall E., Alam A., Perrone R.D. (2019). Autosomal dominant polycystic kidney disease. Lancet.

[2] Harris P.C., Torres V.E. (2009). Polycystic kidney disease. Annu. Rev. Med..

[3] Boucher C., Sandford R. (2004). Autosomal dominant polycystic kidney disease (ADPKD, MIM 173900, PKD1 and PKD2 genes, protein products known as polycystin-1 and polycystin-2). Eur. J. Hum. Genet..

[4] Peces R., Drenth. J.P., Te Morsche R.H., González P., Peces C. (2005). Autosomal dominant polycystic liver disease in a family without polycystic kidney disease associated with a novel missense protein kinase C substrate 80K-H mutation. World J. Gastroenterol..

[5] Vora N., Perrone R., Bianchi D.W. (2008). Reproductive issues for adults with autosomal dominant polycystic kidney disease. Am. J. Kidney Dis..

[6] Torra R., Sarquella J., Calabia J., Martí J., Ars E., Fernández-Llama P., Ballarin J. (2008). Prevalence of cysts in seminal tract and abnormal semen parameters in patients with autosomal dominant polycystic kidney disease. Clin. J. Am. Soc. Nephrol..

[7] Verlinsky Y., Rechitsky S., Verlinsky O., Ozen S., Beck R., Kuliev A. (2004). Preimplantation genetic diagnosis for polycystic kidney disease. Fertil Steril.

[8] Murphy E.L., Droher M.L., Di Maio M.S., Dahl N.K.S., Dahl N.K. (2018). Preimplantation Genetic Diagnosis Counseling in Autosomal Dominant Polycystic Kidney Disease. Am. J. Kidney Dis..

[9] Torra R., Ars E. (2011). Molecular diagnosis of autosomal dominant polycystic kidney disease. Nefrologia.

[10] Swift O., Vilar E., Rahman B., Side L., Gale D.P. (2016). Attitudes in Patients with Autosomal Dominant Polycystic Kidney Disease Toward Prenatal Diagnosis and Preimplantation Genetic Diagnosis. Genet. Test. Mol. Biomark..

[11] De Rycke M., Goossens V., Kokkali G., Meijer-Hoogeveen M., Coonen E., Moutou C. (2017). ESHRE PGD Consortium data collection XIV-XV: Cycles from January 2011 to December 2012 with pregnancy follow-up to October 2013. Hum. Reprod..

[12] Berckmoes V., Verdyck P., De Becker P., De Vos A., Verheyen G., Van der Niepen P., Verpoest W., Liebaers I., Bonduelle M., Keymolen K. (2019). Factors influencing the clinical outcome of preimplantation genetic testing for polycystic kidney disease. Hum. Reprod..

[13] Chamayou S., Sicali M., Lombardo D., Alecci C., Ragolia C., Maglia E., Liprino A., Cardea C., Storaci G., Romano S. (2020). Universal strategy for preimplantation genetic testing for cystic fibrosis based on next generation sequencing. J. Assist. Reprod. Genet..

[14] Morales-García A.I., Martínez-Atienza M., García-Valverde M., Fontes-Jiménez J., Martínez-Morcillo A., Esteban de la Rosa M.A., de Diego Fernández P., García González M., Fernández Castillo R., Argüelles Toledo I. (2018). Overview of autosomal dominant polycystic kidney disease in the south of Spain. Nefrologia.

[15] Franasiak J.M., Forman E.J., Hong K.H., Werner M.D., Upham K.M., Treff N.R., Scott R.T. (2014). The nature of aneuploidy with increasing age of the female partner: A review of 15,169 consecutive trophectoderm biopsies evaluated with comprehensive chromosomal screening. Fertil. Steril..

[16] Rubio C., Rodrigo L., Garcia-Pascual C., Peinado V., Campos-Galindo I., Garcia-Herrero S., Simón C. (2019). Clinical application of embryo aneuploidy testing by next-generation sequencing. Biol. Reprod..

[17] Reig A., Franasiak J., Scott R.T., Seli E. (2020). The impact of age beyond ploidy: Outcome data from 8175 euploid single embryo transfers. J. Assist. Reprod. Genet..

[18] Kort J., McCoy R., Demko Z. (2018). Lathi, RB. Are blastocyst aneuploidy rates different between fertile and infertile populations?. J. Assist. Reprod. Genet..

[19] Rodrigo L., Meseguer M., Mateu E., Mercader A., Peinado V., Bori L., Campos-Galindo I., Milán M., García-Herrero S., Simón C. (2019). Sperm chromosomal abnormalities and their contribution to human embryo aneuploidy. Biol. Reprod..

[20] Kahraman S., Sahin Y., Yelke H., Kumtepe Y., Tufekci M., Yapan C., Yesil M., Cetinkaya M. (2020). High rates of aneuploidy, mosaicism and abnormal morphokinetic development in cases with low sperm concentration. J. Assist. Reprod. Genet..

[21] Obradors A., Fernández E., Oliver-Bonet M., Rius M., de la Fuente A., Wells D., Benet J., Navarro J. (2008). Birth of a healthy boy after a double factor PGD in a couple carrying a genetic disease and at risk for aneuploidy: Case report. Hum. Reprod..

[22] Minasi M.G., Fiorentino F., Ruberti A., Biricik A., Cursio E., Cotroneo E., Varricchio M.T., Surdo M., Spinella F., Greco E. (2017). Genetic diseases and aneuploidies can be detected with a single blastocyst biopsy: A successful clinical approach. Hum. Reprod..

[23] Del Rey J., Vidal F., Ramírez L., Borràs N., Corrales I., Garcia I., Martinez-Pasarell O., Fernandez S.F., Garcia-Cruz R., Pujol A. (2018). Novel Double Factor PGT strategy analyzing blastocyst stage embryos in a single NGS procedure. PLoS ONE.

[24] Yang X.Y., Li T., Liu X.J., Shen J.D., Cui Y.G., Zhang G.R., Liu J.Y. (2018). Preimplantation genetic diagnosis for infertile males with autosomal dominant polycystic kidney disease. Zhonghua Nan Ke Xue.

[25] García-Pascual C.M., Navarro-Sánchez L., Navarro R., Martínez L., Jiménez J., Rodrigo L., Simón C., Rubio C. (2020). Optimized NGS approach for detection of aneuploidies and mosaicism in PGT-A and PGT-SR. Genes.

[26] Neal S.A., Morin S.J., Franasiak J.M., Goodman L.R., Juneau C.R., Forman E.J., Werner M.D., Scott R.T. (2018). Preimplantation genetic testing for aneuploidy is cost-effective, shortens treatment time, and reduces the risk of failed embryo transfer and clinical miscarriage. Fert. Stert..

